# Radical pancreaticoduodenectomy in elderly patients: a feasible and adequate therapeutic option?

**DOI:** 10.1186/1471-2482-13-S1-A23

**Published:** 2013-09-16

**Authors:** Marcello Garavoglia, Alberto Oldani, Clemente De Rosa, Manuela Monni, Alfonso Terrone

**Affiliations:** 1 Department of Surgery University of Eastern Piedmont “Amedeo Avogadro – Hospital “Maggiore della Carità” Novara, Italy

## Introduction

Pancreatic cancer remains one of the deadliest malignancies in the western emisphere despite improved surgical technique, chemotherapy and radiation therapy [1].

Due to the late diagnosis of pancreatic cancer, surgery is the gold standard method to increase the patients’ overall survival; pancreaticoduodenectomy (or Whipple procedure), with lymph nodes dissection, is the standardized radical surgical procedure for pancreatic and periampullary tumors; it comprehends the removal of pancreas head, distal common bile duct, duodenum and the distal portion of the stomach (if the pylorus preserving technique is not used) and consequently the reconstruction with gastro-jejunal, bilio-jejunal anasthomosis, and Wirsung-gastric or Wirsung-jejunal anasthomosis [2].

Pancreaticoduodenectomy is a complex high risk procedure that has infrequently performed until the 1980s, due to high mortality and morbidity rates; in the last 2 decades, surgical techniques improved and postoperative adverse events became more infrequent; Cameron et al reported, on a series of 1000 consecutive pancreaticoduodenctomies, a mortality and morbidity rates of 1% and 4% respectively [3].

The aim of our study is to consider pancreatic surgery in a population of elderly patients, in order to evaluate mortality, morbidity and outcome.

## Methods

From 01/01/1994 to 01/01/2013, 54 patients, 33 males (61.11%) and 21 females (38.89%) aged more than 70 years old (mean age 73 years, range 70-82), underwent radical pancreaticoduodenectoy for periampullar cancer.

Tumor localization was: pancreatic head in 32 cases (%), Vater ampulla in 11 (20.37%), distal common bile duct in 6 (11.11%), duodenum in 4 (7.41%).

In one case (1.85%), pancreaticoduodenectomy was performed because of pancreatic head infiltration by right colon cancer.

## Results

All patients underwent pancreaticoduodenectomy with Whipple-Child technique; macroscopic complete tumor ablation was always achieved.

Standard lymphadenectomy was performed in 43 cases (79.63%), extended lymphadenectomy in 7 (12.96%).

Mucosa-to-mucosa Wirsung-jejunal anastomosis was chosen in 34 cases (62.96%); 19 patients (35.19%) were reconstructed with Wirsung-gastric anastomosis; in 1 case no pancreatic duct reconstruction was performed.

Perioperative mortality rate was 3.70%; one patient died for postoperative acute pancreatitis and one for respiratory failure.

Morbidity was 14.81%; 2 patients (3.70%) developed postoperative peritoneal bleeding; we have also observed 3 cases of gastrointestinal bleeding (5.56%), portal thrombosis in 2 patients (3.70%) and one pancreatic fistula (1.85%).

All postoperative complications have been managed conservatively except peritoneal bleedings, in which re – operation has been necessary.

No differences in terms of morbidity have been demonstrated between the two methods of reconstruction (Wirsung-gastric versus Wirsung-jejunal anasthomosis); Wirsung-jejunal anasthomosis seems to prevent pancreatic atrophy in long surviving patients.

Overall 3 years and 5 years survival was respectively 22% and 15%.

3 and 5 years survival rates were 24% and 16% in patients without lymph node involvement, 2% in patients with lymph node metastatization.

Causes of death have been liver metastasis (25.97%), local recurrence (11.11%), cardiovascular event (3.70%).

## Conclusions

The considerable increase of the aged population in western civilization within the next years will result in a rising incidence of pancreatic cancer. Until the year 2020 an increment of 20% of patients beyond 65 years old can be anticipated; until now only a few retrospective data analyses evaluating the perioperative and long term outcome in geriatric patients exist [4].

Our experience shows that radical surgery for periampullar malignancies is a good option in elderly patients; despite comorbidities we observed good results in terms of survival, mortality and morbidity, similar to what observed in younger patients.
